# Cutaneous leukocytoclastic vasculitis associated with verapamil and atorvastatin: A case report

**DOI:** 10.1002/ccr3.7683

**Published:** 2023-07-17

**Authors:** Yi Tong Vincent Aw, Jonathan Thomas McGuane

**Affiliations:** ^1^ General Medicine Department, Canberra Hospital Canberra Health Services Canberra Australia; ^2^ Australian National University Medical School Australian National University Canberra Australia; ^3^ ACT Pathology, Canberra Hospital Canberra Health Services Canberra Australia

**Keywords:** atorvastatin, cryoglobulinemia, leukocytoclastic, Sjogren's, vasculitis, verapamil

## Abstract

We report a case of biopsy‐proven cutaneous leukocytoclastic vasculitis developing 10 days after starting verapamil and atorvastatin in a patient with long‐standing Sjogren's syndrome. This highlights the need to monitor for this rare adverse effect.

## Graphical abstract

Histopathological examination of purpuric rash. (A) Hematoxylin and eosin staining demonstrating leukocytoclastic vasculitis of small vessels in the superficial dermis (arrows). Positive direct immunofluorescence staining for (B) IgM and (C) C3 in the vessel walls (arrows). (D) Negative IgA staining of vessels and perivascular tissues (arrows) showing only non‐specific background signal from dermal collagen. Scale bar as depicted in bottom right corner of each panel.

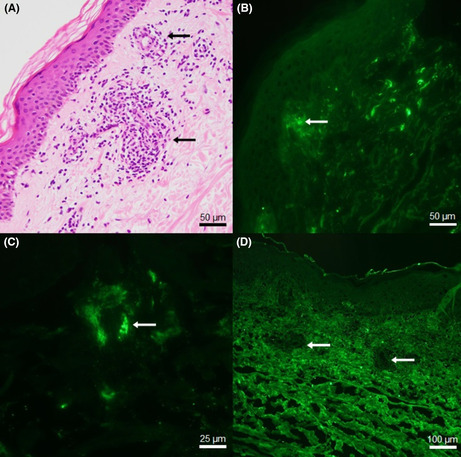

## 1 INTRODUCTION

Vasculitis is an inflammatory disease with variable end‐organ damage that is classified based on involvement of small, medium or large vessels.^1^ Leukocytoclastic vasculitis is a small‐vessel vasculitis predominantly affecting dermal capillaries and venules, and often secondary to underlying systemic vasculitis, infection, or drug exposure.^1^ Commonly implicated drugs include beta‐lactam antibiotics and nonsteroidal anti‐inflammatory drugs,^1^ while calcium channel blockers and hydroxymethylglutaryl‐coA reductase inhibitors (statins) have been rarely associated^2^, ^3^, ^4^, ^5^, ^6^—with no previous formal case report for verapamil. We present a case of cutaneous leukocytoclastic vasculitis associated with recently initiated verapamil and atorvastatin.

## 2 CASE PRESENTATION

**FIGURE 1:**
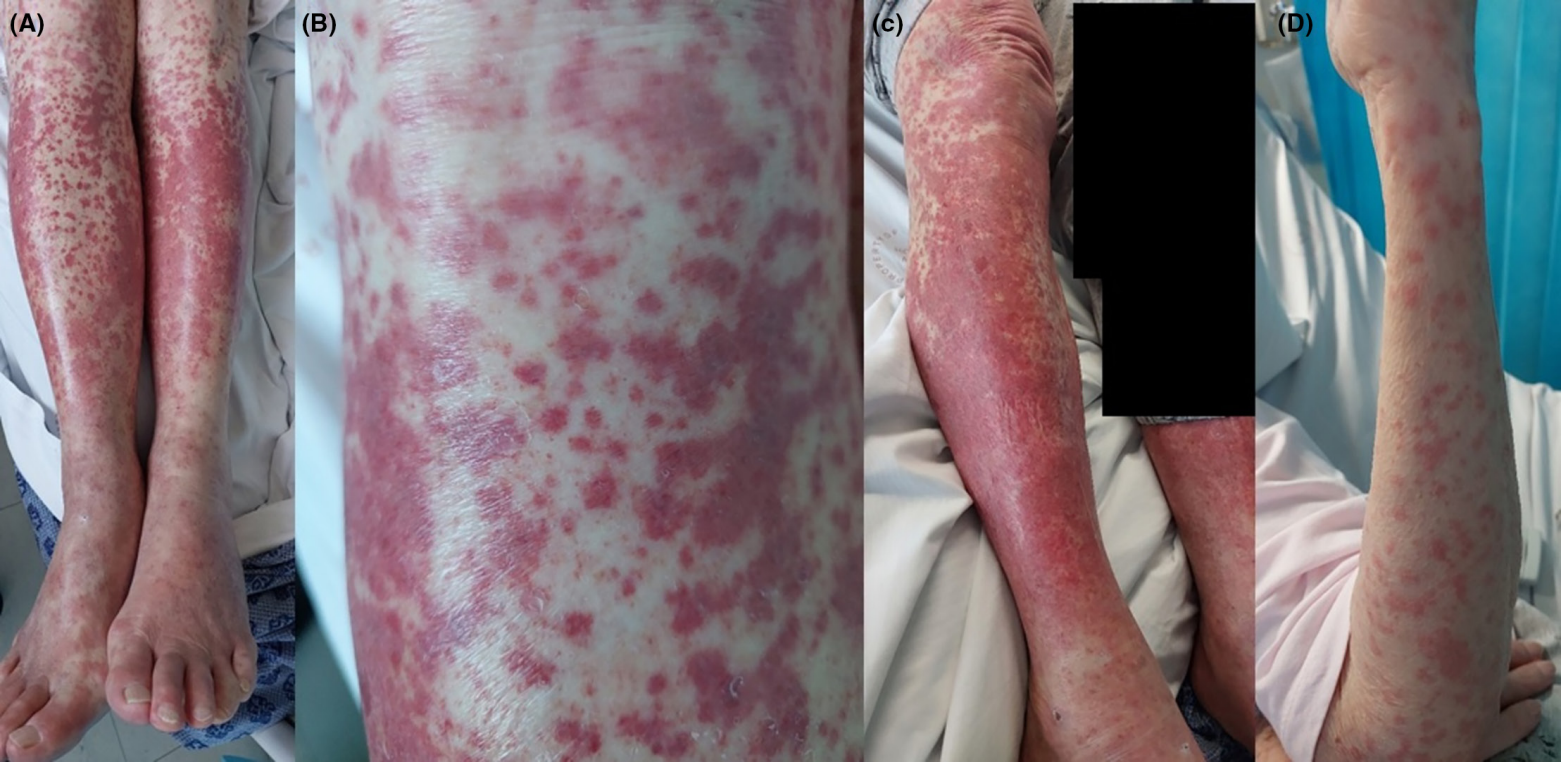
Clinical images of purpuric rash. (A) Bilateral lower limb involvement observed on first day of hospital admission. (B) Close‐up view of purpura. Progression of rash with (C) coalescing of lesions and (D) upper limb involvement by second day of hospital admission. Black boxes placed to obscure clothing pattern.

An 82‐year‐old woman presented to hospital with a 2‐day history of a bilateral lower limb purpuric rash starting over her distal calves with ascent to her thighs (Figure 1A,B). The rash was non‐pruritic and mildly tender. She had no fevers, myalgia, arthralgia, oral or genital ulcers, respiratory or sinus symptoms, neurological symptoms, Raynaud's phenomenon or esophageal dysmotility. She did not have a previously similar rash. She was otherwise well with no preceding infective symptoms. She did not have direct skin contact with allergenic materials. Ten days prior to rash onset, she was commenced on verapamil 40 mg immediate‐release tablet twice daily and atorvastatin 40 mg tablet nocte by her cardiologist for palpitations and dyslipidemia. She took these medications as prescribed for 12 consecutive days until hospital presentation. She had never taken these medications previously.

Her medical history included Sjogren's syndrome diagnosed 3 years prior with high‐titre centromere pattern anti‐nuclear antibodies (ANA) and sicca symptoms—which were stable on long‐term hydroxychloroquine 200 mg once daily treatment. She also had a left submandibular gland Mucosa‐associated lymphoid tissue (MALT) lymphoma successfully excised 1 year prior and was since in remission without needing systemic therapy. Her long‐term medications also included aspirin 100 mg once daily for primary prevention, and over‐the‐counter magnesium, fish oil, and vitamin C supplements taken as per directions. She had no known allergies. Vaccinations were up to date including four SARS‐CoV‐2 vaccinations—receiving the last vaccine 6 months prior without any adverse effects. She had never smoked or taken any illicit drugs and had no alcohol consumption.

On examination, her observations were normal and she was afebrile. She had bilateral lower limb purpura from distal calves to proximal thighs. Over the course of the 2‐day admission, the purpura progressed with coalescing of lesions and ascending involvement of the trunk and upper limbs (Figure 1C,D). She did not have active tenosynovitis, deforming polyarthropathy or nail abnormalities. She had no stigmata of scleroderma or infective endocarditis. She had no ocular inflammation, spondyloarthropathy, aphthous ulcers, or other abnormal rashes. She had no proximal weakness or focal neurological deficits. Her cardiorespiratory examination was unremarkable and she was euvolemic. Her abdominal examination was unremarkable with no hepatosplenomegaly. She did not have significant lymphadenopathy in the cervical, axillary, or inguinal regions.

Her chest x‐ray was unremarkable with no consolidation, interstitial markings, or cardiomegaly. Full blood count revealed a mild normocytic anemia in keeping with anemia of chronic disease, as well as a mild neutrophilia, lymphopenia and monocytosis in keeping with an activated immune response (Table 1). There was no eosinophilia. Platelet count and coagulation profile were normal. C‐reactive protein and erythrocyte sedimentation rate were mildly elevated and high normal respectively; mild hypoalbuminemia and decreased transferrin saturation were present in keeping with an acute inflammatory state. Serum electrolytes, urea, creatinine, liver function tests, and hematinics were unremarkable. Urine investigations revealed no detectable proteinuria, dysmorphic red cells or casts. Infectious serology was negative for hepatitis B, hepatitis C and human immunodeficiency virus (Table 1).

**TABLE 1:** Relevant blood and urine investigations.

Laboratory test	Result	Reference range
Autoimmune serology investigations		
Cryoglobulin	Detected – 0.26 g/L, Type II (Polyclonal + Monoclonal IgM kappa and IgM lambda)	Not detected
C3	0.65 g/L	0.76–1.61 g/L
C4	<0.04 g/L	0.13–0.40 g/L
Rheumatoid factor (RF)	74 IU/mL	<20 IU/mL
Anti‐cyclic citrullinated peptide (Anti‐CCP)	<1 IU/mL	<1 IU/mL
Anti‐nuclear antibodies (ANA)	1: 5120 centromere pattern	Not detected at 1:80
Extractable nuclear antigen antibody screen (ENA)	Not detected for Anti‐SS‐A/Ro60, Anti‐Ro52, Anti‐SS‐B, Anti‐Sm, Anti‐RNP, Anti‐Scl‐70 and Anti‐Jo‐1	Not detected
Anti‐double stranded DNA	<10 IU/mL	Not detected
Anti‐neutrophil cytoplasmic antibodies (ANCA)	Not detected	Not detected
Immunoglobulin A	2.61 g/L	0.76–3.89 g/L
Immunoglobulin M	1.3 g/L	0.3–2.3 g/L
Immunoglobulin G	8.9 g/L	6.5–15.2 g/L
Protein electrophoresis	No paraprotein detected	No paraprotein detected
Total protein	58 g/L	60–80 g/L
Albumin	30 g/L	33–50 g/L
Alpha‐1 globulin	3 g/L	2–4 g/L
Alpha‐2 globulin	9 g/L	5–11 g/L
Beta globulin	6 g/L	5–12 g/L
Gamma globulin	10 g/L	8–16 g/L
Infectious serology investigations		
Hepatitis B surface antigen	Not detected	Not detected
Hepatitis B surface antibody	<10.0 mIU/mL	<10.0 mIU/mL if no previous infection or unvaccinated
Hepatitis B core antibody	Not detected	Not detected
Hepatitis C antibody	Not detected	Not detected
Human immunodeficiency virus	Not detected	Not detected
Hematology investigations		
Erythrocyte sedimentation rate (ESR)	39 mm/h	0–40 mm/h
Hemoglobin	126 g/L	115–160 g/L
Mean cell volume	90 fL	80–96 fL
Platelet count	170 × 10^9^/L	150–400 × 10^9^/L
White cell count	9.2 × 10^9^/L	4.0–11.0 × 10^9^/L
Neutrophil count	7.54 × 10^9^/L	1.8–7.5 × 10^9^/L
Lymphocyte count	0.55 × 10^9^/L	1.2–4.0 × 10^9^/L
Monocyte count	1.01 × 10^9^/L	0.1–1.0 × 10^9^/L
Eosinophil count	0.09 × 10^9^/L	0.0–0.7 × 10^9^/L
Basophil count	0.00 × 10^9^/L	0.0–0.2 × 10^9^/L
Prothrombin time	12 s	12–15 s
Activated partial thromboplastin time	28 s	25–35 s
Serum chemistry investigations		
Sodium	134 mmol/L	135–145 mmol/L
Potassium	4.2 mmol/L	3.5–5.2 mmol/L
Chloride	102 mmol/L	95–110 mmol/L
Bicarbonate	24 mmol/L	22–32 mmol/L
Glucose	6.3 mmol/L	3.5–5.4 mmol/L
Urea	8.2 mmol/L	3.4–9.0 mmol/L
Creatinine	66 μmol/L	45–90 μmol/L
Estimated glomerular filtration rate (eGFR)	75 mL/min/1.73m^2^	>90 mL/min/1.73m^2^
Osmolality	282 mOsm/kg	280–300 mOsm/kg
C‐reactive protein (CRP)	7.6 mg/L	<5.0 mg/L
Bilirubin	13 μmol/L	<20 μmol/L
Alanine aminotransferase	32 U/L	<40 U/L
Alkaline phosphatase	70	30–110 U/L
Gamma glutamyltransferase	29	<50 U/L
Thyroid‐stimulating hormone (TSH)	1.07 mIU/L	0.34–3.40 mIU/L
Vitamin B12	581 pmol/L	120–680 pmol/L
Folate	25.5 nmol/L	7–40 nmol/L
Ferritin	69 μg/L	15–200 μg/L
Transferrin saturation	7%	15%–50%
Urine investigations		
Urine protein	<0.07 g/L	<0.07 g/L
Urine creatinine	3.2 mmol/L	1.1–20.3 mmol/L
Urine leukocytes	<10 × 10^6^/L	<10 × 10^6^/L
Urine erythrocytes	<10 × 10^6^/L	<10 × 10^6^/L
Urine casts or dysmorphic red cells	Nil	Nil
Urine culture	No culture growth after 48 h	No culture growth after 48 h

Abbreviations: C3: complement component 3; C4: complement component 4; DNA: deoxyribonucleic acid; Jo‐1: histidyl transfer ribonucleic acid synthetase; RNP: ribonucleoprotein; Ro52: recombinant Ro52; Ro60: recombinant Ro60; Scl‐70: topoisomerase I; Sm: Smith antigen; SS‐A: Sjogren's‐syndrome‐related antigen A; SS‐B: Sjogren's‐syndrome‐related antigen B.

Autoimmune serology was significant for a high‐titre centromere pattern ANA, with negative extractable nuclear antigen antibody screen (ENA). Type II cryoglobulin was detected with a polyclonal and monoclonal immunoglobulin M (IgM) component (Table 1). Correspondingly, rheumatoid factor (RF) was elevated and there was marked C4 hypocomplementemia. Cryoglobulin status was unknown prior to presentation. Anti‐double stranded deoxyribonucleic acid (anti‐dsDNA), anti‐cyclic citrullinated peptide (Anti‐CCP) and anti‐neutrophil cytoplasmic antibodies (ANCA) were negative. Serum immunoglobulin G (IgG), A (IgA) and IgM were within normal range. Serum protein electrophoresis was unremarkable with no paraprotein (Table 1).

**FIGURE 2:**
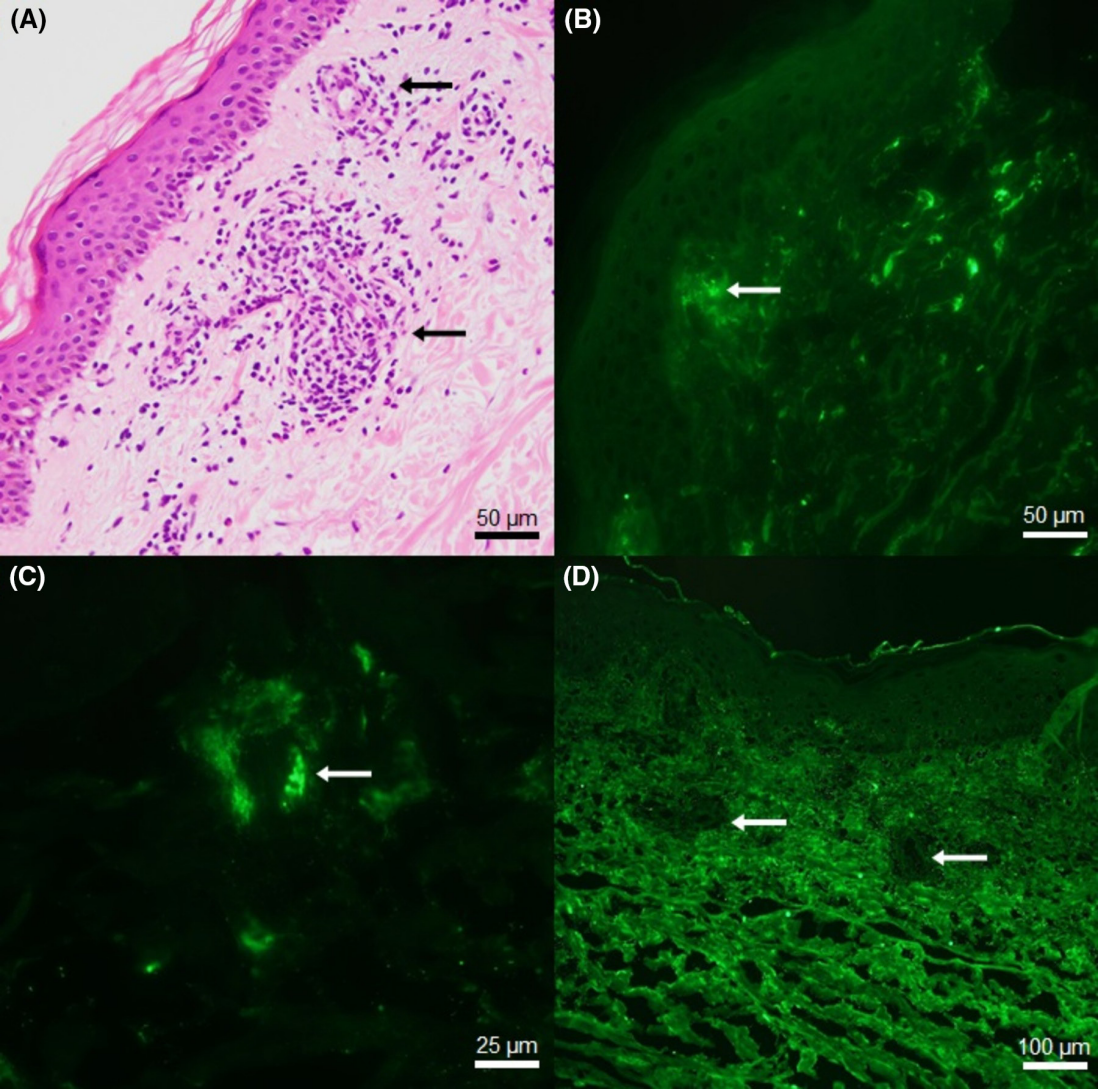
Histopathological examination of purpuric rash. (A) Hematoxylin and eosin staining demonstrating leukocytoclastic vasculitis of small vessels in the superficial dermis (arrows). Positive direct immunofluorescence staining for (B) IgM and (C) C3 in the vessel walls (arrows). (D) Negative IgA staining of vessels and perivascular tissues (arrows) showing only non‐specific background signal from dermal collagen. Negative staining also observed for IgG (not shown). Scale bar as depicted in bottom right corner of each panel.

Histopathology of the purpuric rash revealed leukocytoclastic vasculitis involving small vessels in the papillary and upper reticular dermis, with endothelial swelling and associated perivascular infiltrate of neutrophils, lymphocytes, few eosinophils, and leukocytoclasis (Figure 2A). Direct immunofluorescence revealed positive staining of the vessel walls for IgM (Figure 2B), C1q and C3 (Figure 2C). Staining for IgG and IgA (Figure 2D) was negative.

Based on the presentation and results, a diagnosis was made of mixed cryoglobulinemia secondary to Sjogren's syndrome, with new onset cutaneous leukocytoclastic vasculitis arising from a differential diagnosis of a drug‐induced type III hypersensitivity reaction and/or flare of cryoglobulinemic vasculitis.

## 3 OUTCOME AND FOLLOW‐UP

Atorvastatin and verapamil were ceased from hospital presentation. Prednisolone 30 mg once daily was commenced and she was discharged with a plan to reduce dosage by 5 mg every 5 days. Upon follow‐up 1 week later, there was marked improvement of the purpuric rash with no residual scarring or pigmentation. On 1 month follow‐up, the rash had resolved and there was no evidence of end‐organ or systemic vasculitis manifestations.

## 4 DISCUSSION

Calcium channel blockers and statins have been rarely associated with leukocytoclastic vasculitis. A literature search of PubMed and Google Scholar using the terms “leukocytoclastic vasculitis”, “cryoglobulin”, “calcium channel blocker” “verapamil”, “statin” and “atorvastatin” yielded only a few similar case reports.^2^, ^3^, ^4^, ^5^, ^6^ Among the calcium channel blockers, only diltiazem,^3^ lercarnidipine,^4^ and amlodipine^5^ have been implicated. We present here a case of leukocytoclastic vasculitis following atorvastatin and verapamil initiation. Notably, leukocytoclastic vasculitis can occur with Sjogren's syndrome without additional inciting causes.^1^ Although possible, we consider this an unlikely explanation in this case given the close temporal relationship between commencing medication and the development of symptoms, and the fact that the patient's long‐standing Sjogren's syndrome was well‐controlled at the time. This caveat notwithstanding, the case scored five points on the Naranjo scale,^7^ making it a “probable” adverse drug reaction. We are not aware of any published cases of leukocytoclastic vasculitis associated with verapamil, while only one other report has previously demonstrated an association with atorvastatin.^2^

Given both medications were started and ceased simultaneously, it is unclear whether the inciting agent was verapamil, atorvastatin, or the combination. It is well recognized that verapamil and atorvastatin interact via CYP3A4 enzyme and P‐glycoprotein inhibition.^8^ In this case, use of this combination may have increased the concentration of either drug to supratherapeutic levels and therefore increased the risk of inducing leukocytoclastic vasculitis—assuming a dose‐dependent mechanism.

We postulate that the leukocytoclastic vasculitis resulted from a type III hypersensitivity reaction—where drug‐induced aberrant antibody production leads to immune complex formation and deposition in small vessels causing vasculitis—thought to be the general mechanism in drug‐induced leukocytoclastic vasculitis.^9^ This is supported by positive IgM and complement staining on immunofluorescence, skin‐limited disease, and rash onset within the typical window of 7–10 days after commencing medications.^1^

However, the patient also had typical features of mixed cryoglobulinemic vasculitis—the purpuric rash with leukocytoclastic vasculitis, C4 hypocomplementemia, elevated RF and type II cryoglobulinemia with polyclonal and monoclonal IgM components—which would also account for positive IgM and C3 staining on immunofluorescence studies. Cryoglobulinemia is most likely secondary to Sjogren's syndrome given the recognized association.^10^ Given the temporal relationship between initiation of the medications and development of leukocytoclastic vasculitis, a second postulated mechanism is drug‐induced augmentation of cryoglobulinemia—leading to an immune complex‐mediated flare of cryoglobulinemic vasculitis.^10^ Based on our literature search, this would be the first reported case of a cryoglobulinemic vasculitis flare associated with atorvastatin or verapamil.

It is possible that both mechanisms contributed in this case. Given the vasculitis was skin‐limited, we favor the former hypersensitivity reaction as the predominant mechanism over a drug‐induced flare of cryoglobulinemic vasculitis—as this typically exhibits joint, renal, and neurological manifestations,^10^ which were absent in this patient.

Future research into molecular mechanisms would be beneficial to better understand the properties of certain medications which mediate autoimmunity and clarify genetic susceptibility to drug‐induced vasculitis.^9^

In summary, we present a case of biopsy‐proven leukocytoclastic vasculitis associated with verapamil and atorvastatin. We postulate the mechanism to be a type III hypersensitivity reaction or a drug‐induced flare of cryoglobulinemic vasculitis secondary to Sjogren's syndrome. With respect to verapamil, this is the first report of this probable adverse drug reaction, and only the second regarding atorvastatin. This case report highlights the need for prescribers and clinicians to carefully consider drug interactions and monitor for rare adverse effects after initiating the common medications of verapamil and atorvastatin—among the most prescribed medicines in Australia^11^—particularly in patients with autoimmune disease.

## AUTHOR CONTRIBUTIONS

**Yi Tong Vincent Aw:** Conceptualization; data curation; formal analysis; writing – original draft; writing – review and editing. **Jonathan Thomas McGuane:** Formal analysis; writing – review and editing.

## FUNDING INFORMATION

The authors declare no financial support.

## CONFLICT OF INTEREST STATEMENT

The authors declare no potential conflicts of interest.

## ETHICS AND PATIENT CONSENT FOR PUBLICATION STATEMENT

Written informed consent was obtained from the patient to produce this case report and clinical images. This research was carried out in accordance with the Declaration of Helsinki.

## Data Availability

Data sharing not applicable to this article as no datasets were generated or analysed during the current study.
